# Synthetic microfiber emissions to land rival those to waterbodies and are growing

**DOI:** 10.1371/journal.pone.0237839

**Published:** 2020-09-16

**Authors:** Jenna Gavigan, Timnit Kefela, Ilan Macadam-Somer, Sangwon Suh, Roland Geyer

**Affiliations:** Bren School of Environmental Science and Management, University of California, Santa Barbara, CA, United States of America; University of Miami, UNITED STATES

## Abstract

Synthetic microfibers are found virtually everywhere in the environment, but emission pathways and quantities are poorly understood. By connecting regionalized global datasets on apparel production, use, and washing with emission and retention rates during washing, wastewater treatment, and sludge management, we estimate that 5.6 Mt of synthetic microfibers were emitted from apparel washing between 1950 and 2016. Half of this amount was emitted during the last decade, with a compound annual growth rate of 12.9%. Waterbodies received 2.9 Mt, while combined emissions to terrestrial environments (1.9 Mt) and landfill (0.6 Mt) were almost as large and are growing. Annual emissions to terrestrial environments (141.9 kt yr^-1^) and landfill (34.6 kt yr^-1^) combined are now exceeding those to waterbodies (167.2 kt yr^-1^). Improving access to wastewater treatment is expected to further shift synthetic microfiber emissions from waterbodies to terrestrial environments. Preventing emissions at the source would therefore be a more effective mitigation measure.

## Introduction

Plastic pollution has emerged as a key environmental issue in recent years. Global production and use of plastics, or synthetic polymers, has grown at a compound annual growth rate of 8.3% since the beginning of mass production around 1950 [1]. Annual plastic production has surpassed the output of most other man-made materials [1]. Half of all plastics ever made by humankind were produced in the last 13 years [2]. Between 1950 and 2017 an estimated 9,200 million metric tonnes (Mt) of virgin plastics were produced, of which 5,300 Mt were discarded in landfills, dumps, or the natural environment [2].

Synthetic fibers make up 14% of global plastics production [2] and can generate synthetic microfibers through fragmentation and degradation [3–8]. Synthetic microfibers are considered a type of microplastic, which is typically defined by the longest dimension being less than 5mm [9]. Microplastics have raised concerns due to their increased bioavailability, impacts on low-trophic organisms through the uptake of toxic chemicals [3,10,11], and increased risk of mortality due to ingestion [12]. Microfibers constitute a significant fraction of microplastics accumulating in freshwater [13,14], marine [10,15], coastal [6,16], terrestrial [17–19], and arctic ecosystems [20] where they pose risks to aquatic organisms and terrestrial biodiversity [12,21–24]. Humans are also impacted by the inhalation of airborne microfibers and consumption of microfibers found in common foods such as water, alcohol, seafood, sugar, and honey [25,26]. Current literature suggests that microfibers may bioaccumulate in the lungs and trigger inflammation, however, the human health impacts are not well understood [27].

Apparel washing has been established as a major source of synthetic microfiber emissions [3–8]. Quantifying the amount of synthetic microfibers emitted to the environment, however, has been challenging due to large gaps in data and the global scale of emissions. Fibers in clothing undergo mechanical fragmentation during the washing process, thereby releasing microfibers [3,28]. Various factors influence microfiber release rates, including garment age [3], washing machine type [3,7], and detergent use [4,5,7]. While previous studies focus on washing machine emissions, a significant fraction of global apparel washing is done by hand, from which lower shed rates are expected as compared to machine washing. Laundry effluent conveys microfibers into wastewater streams and is either processed by wastewater treatment plants (WWTPs) or emitted directly into the natural environment [6]. Wastewater treatment plants with preliminary, primary, and secondary treatment can remove up to 98–99% of microfibers which are then retained in WWTP biosolids [29–37]. Biosolids are commonly used as soil amendments, providing a route for synthetic microfibers into terrestrial environments where they can remain detectable in soils for up to fifteen years after application [17,18,38]. Microfibers that are not removed during treatment typically fall within the smallest size range and are ejected into receiving fresh or marine waterbodies [33].

This study presents a global material flow analysis of synthetic microfiber release from apparel washing covering the period from 1950 to 2016, i.e. from the beginning of mass production of synthetic fibers to the present. We take a new approach, using time-dependent modeling of regional in-use stocks of synthetic fibers in apparel and shed rates during the washing process that distinguish between hand and machine washing by region. Furthermore, we compile regional wastewater treatment data in order to estimate the quantity of microfibers that end up in untreated wastewater, treated effluents, and biosolids from treatment plants. The final step incorporates biosolid management data and estimates how much of the synthetic microfibers from apparel washing enters four initial compartments: fresh and marine waterbodies, terrestrial environments, landfills, and incineration (S1 Fig in S1 File). For this analysis, we group fresh and marine waterbodies into one category which will be referred to as “waterbodies” throughout the text below.

## Methods

Our calculations were based on: (i) global apparel production by year, (ii) regionalized global consumption and in-use stock estimates, (iii) regionalized end-of-life apparel estimates, (iv) estimates of microfiber emissions from apparel washing, and (v) the fate of microfiber emitted from washing considering the regional share of wastewater treated and the fate of sludge from wastewater treatment plants (S1 Fig in S1 File). What follows is an elaboration of each step.

### Annual global apparel production

Global synthetic fiber production data from 1980 to 2016 were generated by reconciling multiple global data sources including The Fiber Year 2017, PCI WoodMackenzie, and Orbichem Final [39–41]. These data sets were combined by taking the mean values. To estimate synthetic fiber production from 1950–1980, synthetic fiber’s share of total plastic production from 1980–1990 was calculated. The share varied between 14% and 16% with an average of 15%. The value of 15% was used to calculated total fiber production as share of total plastic production for 1950–1980 from existing data [1]. Each fiber type’s average share of total fiber production from 1980–1990 was calculated and showed little variation. The average fiber type shares were used to calculate the amount of each fiber type produced from our total fiber production data from 1950–1980 (S1 Data in S2 File).

Next, we estimated the mass of synthetic fibers used for apparel production (S1 Data in S2 File). Market segmentation data for polyester and nylon were used as a proxy to represent the market segmentation of all synthetic fibers from 1990 to 2016 [41]. We then used linear regression to estimate the market segmentation between 1950 and 1990. To differentiate between fiber types, we set the mass of each fiber type present in apparel to be proportional to the mass of each fiber type produced annually (S1 Data in S2 File). During apparel production, fiber losses occur from fabric cutting, trimming, and mistakes [42]. The amount of fiber lost during the apparel production process is estimated to be 10% to 15% of all fiber used [43,44]. For this study, the value for the amount of fiber lost during apparel production was set at 20% in order to keep the final results conservative. A sensitivity analysis was conducted in order to test the impact of this parameter on our results (see S1 File.).

### Regional consumption and in-use stock estimates

This analysis divides the world into the following regions: North America, Western Europe, Eastern Europe, South and South East Asia, China, and the rest of the world (ROW) which encompasses Latin America, Africa, the Middle East, and Australasia. Time-series regional apparel demand data, which are not publicly available, were acquired from a market research company and were cross-checked with publicly available total production values [41]. These data spanned years 1990–2017 with projections to year 2030. We were unable to access any regional time-series apparel demand data of this sort prior to year 1990, nor were we able to make informed estimations of regional demand trends prior to year 1990. For these reasons, the percentages reported in year 1990, our nearest data point, were held constant and projected backwards to estimate each region’s apparel demand for years 1950 to 1989 (S2 Data in S2 File).

### Apparel reaching its end of life

Apparel use was characterized by a discretized log-normal distribution, LTD(*j*), which denotes the fraction of synthetic fibers in apparel used for *j* years [45] (S1 Data in S2 File). The mean lifetime value of clothing is set at 5 years with a standard deviation (SD) of 1.5 [46]. The total amount of synthetic fiber waste in end-of-life apparel *AW* generated in year *t* was calculated as:

$$AW(t)=\sum_{j=1}^{67}AP(t-j)\times LTD(j)$$

In this above equation, AP is apparel production.

We then calculated the global annual in-use stock of synthetic fibers in apparel *AiS*(*t*) by adding annual apparel production and subtracting annual apparel waste from the in-use stock of the previous year (S1 Data in S2 File):

AiS(t)=AiS(t−1)+AP(t)−AW(t)

### Machine vs. hand washing

To determine the amount of apparel hand washed vs. machine washed each year, we adopted a washing machine technology diffusion model to determine the percentage of households in each region that owned washing machines [47] (S3 Data in S2 File). The results produced by this model were calibrated with existing washing machine ownership data found in literature by adjusting the input parameters for each country to fit the country-level washing machine ownership data available [48–54]. This diffusion relationship follows a logistic function given by:

$${Diff}_{c}=\frac{\alpha }{1+\gamma \;e^{\left(\beta_{inc}I_{c}+\beta_{elec}E_{c}\right)}e}+\varepsilon$$

For this equation: *Diff*_*c*_ is the diffusion of the appliance for country *c*, *α* is the saturation level, which is equal to 1 in the case of washing machines [47], *I*_*c*_ is the household income given by GDP divided by the number of households in the country [55,56], *E*_*c*_ is the electrification rate [57], and *ε* is the error term which is 3.5% for washing machines [47]. The original parameter values are *β*_*inc*_ = -3.5E-05, *β*_*elec*_ = -8.98, and ln γ = 8.91 [47] which were adjusted in such a way that the sum of distances between the available washing machine ownership data points and the logistic function is minimized for each country.

### Washing frequency

We created an annual washing frequency model for the top 30 consuming countries by comparing each country’s washing capacity to their washable stock. The washable stock represents the fraction of clothing in a given country that is active, meaning it has been worn within the past 12 months and is washed. According to published literature, 28–30% of in-use apparel is considered inactive [58]. To keep our estimates conservative, we assumed that 25% of clothing is inactive. A sensitivity analysis was conducted for the percentage of clothing that is inactive (see S1 File). We took the mean washing frequency value across all 30 countries to generate one universal washing frequency value denoted by *f*. While the washing frequency model focuses on machine washing only, we applied the same washing frequency value for machine washing and hand washing.

The washing frequency for country *c*, (*f*_*c*_), was calculated as:

$$f_{c}=\frac{H_{c}\times W_{c}\times L_{c}}{T_{c}\times 0.75}$$

The numerator of this equation represents the washing capacity of country *c*; *H*_*c*_ represents the number of households in country *c* that own washing machines, *W*_*c*_ is the number of washes per households per year [58], and *L*_*c*_ is the average load capacity [58] of country *c*. In the denominator, *T*_*c*_ is the in-use stock of country *c* that is washed by washing machine (S2 Data in S2 File), and 0.75 is the fraction of apparel that is considered active and able to be washed.

### Microfiber shedding during washing

Microfiber shed rates from washing machines were gathered from published literature that reported microfiber shedding in units of mass lost per total mass of garment [3,6]. Determining the average microfiber mass loss of machine-washed garments indicates that microfibers shed at a rate of 0.34 kg per tonne washed [3,4,8,59]. Hand washing shed rates were unavailable due to lack of data. For this reason, we set the hand washing shed rate at 50% of the machine washing shed rate and conducted a sensitivity analysis with a hand wash shed rate that was 25% and 75% of the machine-washing shed rate (see S1 File).

The mass of microfibers shed for each country was calculated as:

$$m_{c}=(S_{c}\times 0.75\times M_{c}\times f\times r_{m})+(S_{c}\times 0.75\times (1-M_{c})\times f\times r_{h})$$

In this equation, *m*_*c*_ is the mass of synthetic microfibers shed in country *c*, *S*_*c*_ is the apparel stock of country *c*, 0.75 is the fraction of the apparel stock that is active, *M*_*c*_ is the percentage of households owning washing machines in country *c*, *f* is the washing frequency, *r*_*m*_ is the machine wash shed rate, *r*_*h*_ is the hand wash shed rate.

### Percentage of laundry effluent treated and untreated

Microfibers shed from washing are conveyed by laundry effluent into the wastewater stream [3,6]. Data reporting the percentage of each country’s residential population connected to wastewater treatment was used as a proxy for the percentage of wastewater treated in each region [60–62]. Synthetic microfibers retained in untreated wastewater were assumed to directly enter waterbodies. Wastewater data was sparsely available for 97 countries between 1990 and 2016 and most countries did not have reported data for the entire 27-year timespan. Out of the 70 countries included in this analysis, 24 had no available wastewater data. To fill the data gaps for countries with two or more data points, the data was interpolated and extrapolated based on detectable trends of wastewater infrastructure. For countries with no available data, all 97 original countries were grouped by income class based on the World Bank’s 2016 income classifications (high income, upper-middle income, lower-middle income, low income). The average annual percentage of population connected to wastewater treatment for each income class was then applied as a proxy for the 24 countries with no available data, a method adopted from Sato et al. (2013) to determine individual country’s use of wastewater [63]. The last estimated or reported value was then extrapolated backwards to extend the temporal coverage to 1950–2016 (S4 Data in S2 File).

### Type of wastewater treatment and fiber retention rate

As documented in the literature, there is a lack of data on the type of treatment treated wastewater receives [63–65]. Because of this, the following assumptions regarding the type of wastewater treatment (i.e. primary, secondary, and tertiary), used by each country were made: 100% secondary for high income countries, 50% secondary and 50% primary for upper-middle income, 25% secondary and 75% primary for lower-middle income, and 100% primary for low income countries. Each country’s population size was then used to produce regional weighted averages. Tertiary treatment was not included in this analysis due to insufficient data regarding its use globally. Microfiber retention rates during primary, secondary, and tertiary treatment were compiled from existing literature (S4 Table in S1 File) [29–37].

### Fate of sludge

Eurostat, Organization for Economic Cooperation and Development (OECD), and country-specific data sets as well as peer-reviewed publications were combined to determine the fate of sludge, i.e. incineration, dumped at sea, landfilled, or applied on terrestrial environments. This was done for years 1970, 1975, 1980, 1985, and 1989–2016 [66–72]. Due to sparse data, these sources reported on 61 countries with no country reporting data for the entire 33-year timeframe described above. As such, the same extrapolation and interpolation methods described in the wastewater treatment segment were applied here. In some instances, the original data reported did not sum to 100%. In these cases, sludge application values were proportionally adjusted so that the sum of all potential sludge applications equaled 100% (S5 Data in S2 File).

## Results

### Synthetic fiber production and in-use stock

Our results show that the global in-use stock of synthetic fiber in apparel increased from 0.10 Mt in 1950 to 196 Mt in 2016 (S1 Data in S2 File). A total of 1,178 Mt of synthetic fibers were produced between 1950 and 2016, of which around 738 Mt were used in apparel production (S1 Data in S2 File). We estimate that in 2016, 43 Mt of synthetic fiber entered the in-use phase of apparel while 34 Mt were discarded, resulting in a net addition of 9 Mt of synthetic fiber to the in-use apparel stock in that year. The 2016 in-use stock of synthetic fiber in apparel for China alone is estimated to be 76 Mt, which accounts for about 40% of the global 2016 synthetic fiber stock (Table 1). If South and South East Asia are combined, Asia as a whole, including China, accounts for 62% of global synthetic fiber stock as apparel in 2016 (Table 1). The amount of in-use stock per capita, however, is the highest in North America where 62 kg of synthetic fiber per capita was held as apparel in 2016 (S5 Table in S1 File). The global average per capita stock increased from 8 kg per capita in 1990 to 26 kg per capita in 2016 with a compound annual growth rate (CAGR) of 5%. China’s per capita in-use stock grew almost twice as fast starting at 5 kg per capita in 1990 and arriving at 51 kg per capita in 2016 with a CAGR of 9%.

**Table 1:** 2016 Regional apparel in use stock in million metric tonnes (Mt), percentage of clothing machine washed, wastewater treatment rate, microfibers shed, and fates of microfibers, both in thousands of tonnes (kt). The four fates of microfibers are: waterbodies, terrestrial environments, landfill, and incineration. ROW stands for rest of world.

Region	Stock in use (Mt)	% machine washed	Wastewater treatment rate	Microfiber shed (kt)	Fate of microfibers (kt)
						Water-bodies	Terrestrial environments	Landfill	Incineration
China	76	100%	50%	157	77	70	9	0
South/SE Asia	44	33%	21%	61	43	16	2	0
North America	25	89%	79%	49	11	22	10	6
West Europe	17	98%	90%	35	6	17	2	9
ROW	20	29%	44%	31	16	9	4	1
East Europe	13	96%	50%	27	12	7	7	1
Total	196	-	-	360	167	142	35	17

### Synthetic microfiber shedding from apparel washing

**Fig 1:**
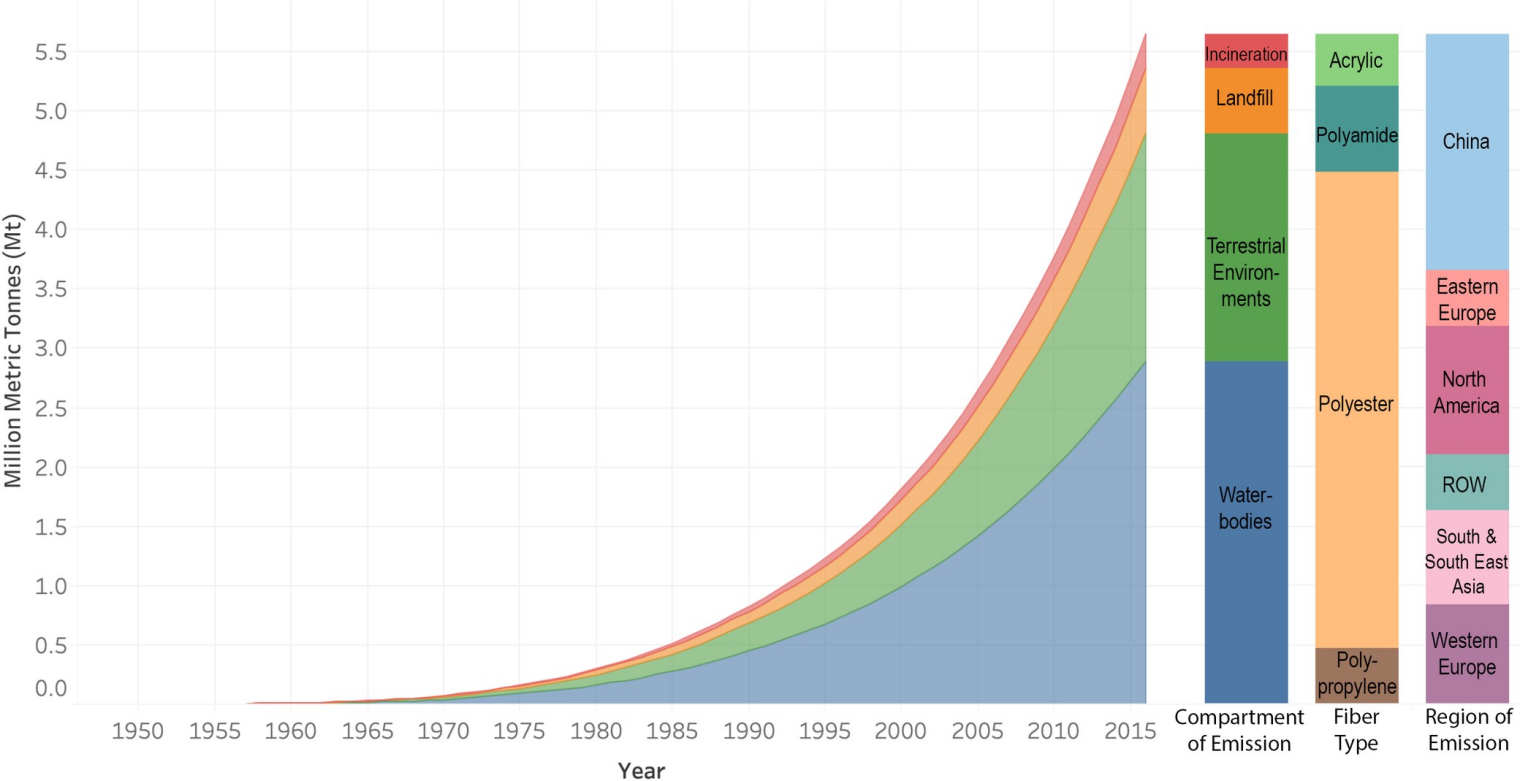
Cumulative microfiber emissions from 1950 to 2016. The right columns describe compartmental, compositional, and regional characteristics of the cumulative mass of microfiber emitted by 2016.

As the global stock of synthetic fiber has grown over the years, the global amount of synthetic microfiber shedding too has increased from around 122 tonnes in 1950 to an estimated 360 kilo tonnes (kt) in 2016, with a CAGR of 12.9%. Synthetic microfiber shedding grew faster than apparel consumption and the synthetic fiber in-use stock, due to the combined effect of consumption growth and higher access to washing machines. Adding year-over-year estimates, a cumulative 5.6 Mt of synthetic microfibers have been shed and emitted from combined hand and machine washing globally between 1950 and 2016. Roughly half of these emissions occurred during the last decade (Fig 1). Approximately 2.9 Mt of the synthetic microfiber emissions entered waterbodies, which is equivalent to over 7 billion fleece jackets by mass. In addition, an estimated 1.9 Mt of synthetic microfibers were applied to terrestrial environments, 0.6 Mt landfilled, and 0.3 Mt incinerated (Fig 1). 88% of all microfiber emissions to waterbodies were from untreated wastewater, 8% from treated wastewater, and 4% from biosolids discarded into waterbodies. 92% of synthetic microfiber emissions to terrestrial environments came from biosolids, while the other 8% were delivered by untreated wastewater used for irrigation. Both landfill and incineration serve as disposal methods for synthetic microfibers retained in biosolids. The fiber type composition of the global cumulative synthetic microfiber emissions is 4.0 Mt polyester, 0.7 Mt polyamide, 0.5 Mt polypropylene and 0.4 Mt acrylic (Fig 1).

### Regional emissions

**Fig 2:**
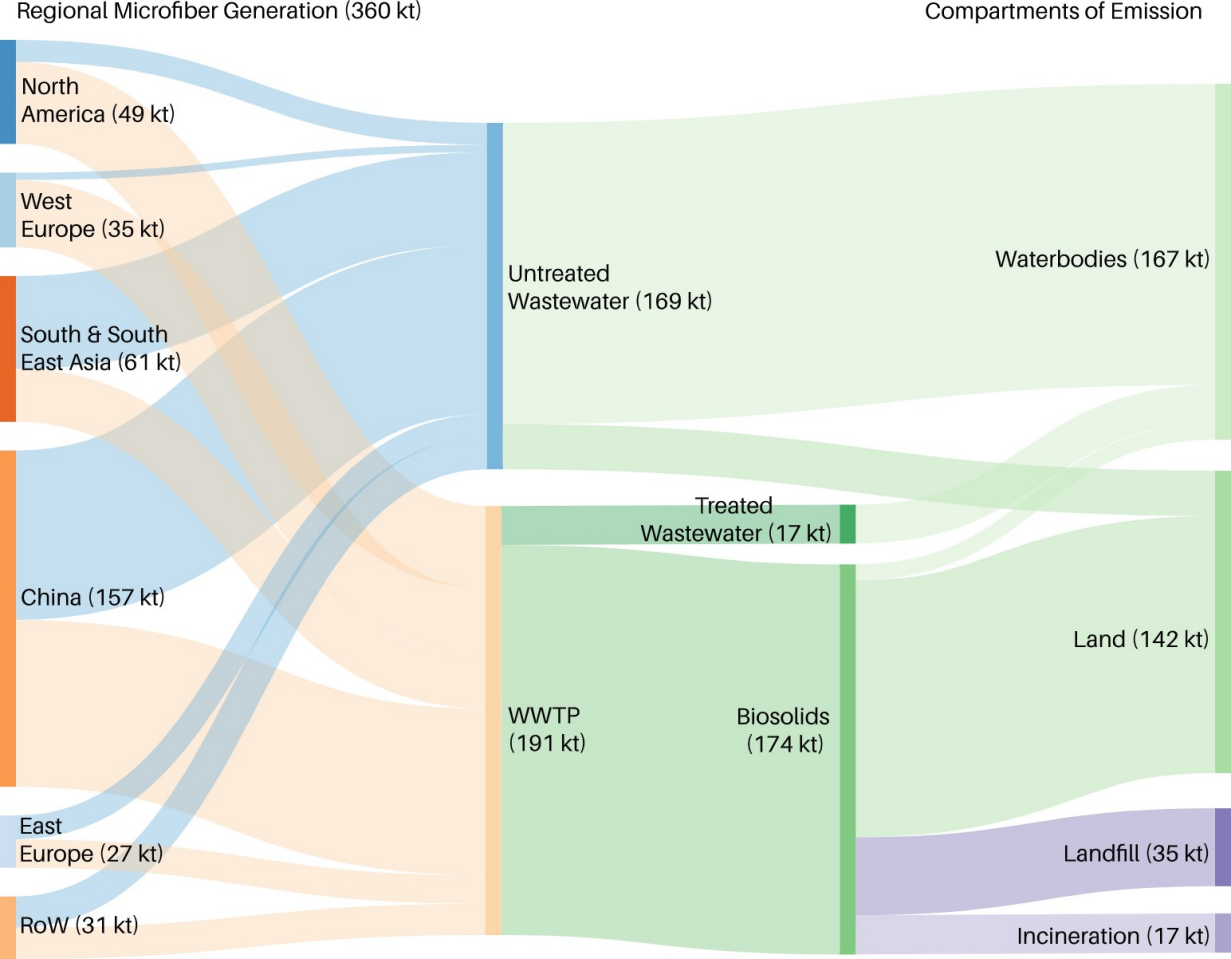
The emission pathways of synthetic microfibers generated from apparel washing in 2016. The leftmost column represents regional microfiber release. The middle columns represent the flow of synthetic microfibers through available infrastructure. The rightmost column represents the initial receiving environmental compartments.

In 2016, China generated 157 kt of synthetic microfiber emissions, and the rest of Asia 61 kt, the largest and second largest quantities of all the regions shown in Table 1. This is primarily due to the large population in Asia and the significant increase in both clothing consumption and washing machine ownership over the past decade (see Table 1). About half of the 157 kt of 2016 synthetic microfiber emissions from apparel washing in China entered waterbodies directly without wastewater treatment (Fig 2). It is estimated that about 47%, or 69 kt, of global microfiber emissions to waterbodies through untreated wastewater came from China and 28%, or 42 kt, from other Asian regions. North America and Western Europe had the third and fourth highest rates of synthetic microfiber release from apparel washing in 2016: 49 kt and 35 kt, respectively. However, most microfiber emissions in these regions entered WWTPs (Fig 2), which reduced their global contribution of microfiber emissions to waterbodies through untreated wastewater to 6% and 2%, respectively. Instead, 45% and 50% of synthetic microfibers generated in North America and West Europe, respectively, were emitted into terrestrial environments due to widespread access to WWTPs (Table 1).

## Discussion & conclusion

Our results indicate that microfiber emissions to the environment are undergoing both quantitative and qualitative changes. In terms of the quantity of emissions, both the global in-use apparel stock and washing machine ownership are increasing, resulting in the release of growing quantities of synthetic microfibers into natural environments. In terms of quality, terrestrial environments are emerging as an increasingly important initial compartment of microfiber emissions, which is due to improved access to WWTPs and the application of biosolids to terrestrial environments (Fig 1). Increases in WWTP coverage in coming decades will therefore not necessarily result in an overall decrease of synthetic microfiber emissions to the environment, but rather divert synthetic microfibers from waterbodies to terrestrial environments and landfills, unless microfibers are removed from sewage sludge prior to land application. Sludge treatment technologies are seen as a potential interventions to remove microplastic particles from sludge before land application [73]. More research is required to determine the efficacy of such microplastic removal technologies. Recent literature suggests that synthetic microfibers can affect physical soil properties and microbial activity which may pose consequences for soil function and plant performance [24,74,75]. Synthetic microfiber contamination in terrestrial environments is an emerging topic and further research is required to understand the implications. Furthermore, the microfibers initially emitted to terrestrial environments have the potential to eventually enter other compartments, including waterbodies and biota, through runoff, resuspension, or convection over a long period of time [76,77]. Little is known as to the transport and fate of microfibers emitted to the environment [76–79].

Due to the nature of the method and data used, multiple assumptions and simplifications were made, which generates considerable uncertainty. Sensitivity analysis was conducted for each parameter included in this study (see S1 File for details). Microfiber emission results are found to be sensitive to five key parameters: washing machine ownership (moderately sensitive), washing frequency, percentage of clothing in-use, microfibers shed rate, and microfiber retention rate in WWTPs (all highly sensitive). Alternative scenarios are tested for these five parameters (see S1 File). These alternative scenarios result in total cumulative microfiber emissions ranging from 4.3 Mt to 7.0 Mt.

Due to the limitations of this study, our results do not represent the total emissions produced from apparel throughout its lifetime. We do not take into consideration apparel that is reused, donated, or recycled. Since there are large markets for secondhand clothing, especially in lower income countries, taking this factor into account would likely increase the stock of in-use apparel and therefore increase emissions. Instead, our current model considers all clothing to be taken out of circulation once reaching its end of life. In addition, fiber and apparel production processes are likely to produce a substantial quantity of microfiber emissions, however, these processes are not accounted for in this study. We also did not account for wastewater overflows during storm events or instances in which WWTPs are over capacity. In these instances, it is likely that a larger quantity of synthetic microfibers enter waterbodies and a smaller quantity enter terrestrial environments via biosolid applications.

This study presents the first global estimate of synthetic microfibers emitted to the environment from apparel washing between 1950 and 2016. The broad scope helps to understand the magnitude and temporal trends of microfiber emissions, and to identify meaningful mitigation measures. Large-scale removal of microfibers from the environment is unlikely to be technically feasible or economically viable, so the focus needs to be on emission prevention. Given that increasing global access to WWTP does not necessarily reduce the microfiber emissions to the total environment, preventing or reducing microfiber emissions before they enter the sewer system should be a priority. This means either reducing the microfiber shed rates from apparel washing or capturing the shed microfibers during the washing process. The former might be achievable through redesign of the washing process, the fiber-containing textiles, or the fibers themselves. The latter would require some type of pollution control technology at the washing stage, such as lint filters or microfiber trapping bags or other devices. In fact, in some countries all washing machines are already equipped with lint filters. Trapped microfibers still require final disposal, such as landfill or incineration. While more research on the interactions between microfibers and the environment is urgently needed, especially in terrestrial ecosystems, this should not stop us from enacting mitigation measures now.

## Supporting information

S1 File (DOCX)

S2 File (XLSX)
