# Supplementary material for: Synthetic microfiber emissions to land rival those to waterbodies and are growing
Source: PLoS One. 2020 Sep 16;15(9):e0237839. doi: 10.1371/journal.pone.0237839 (PMC7494121; doi:10.1371/journal.pone.0237839)
Supplement: S1 File — (DOCX) [file pone.0237839.s001.docx]

**Sensitivity Analysis**

This sensitivity analysis measures the reactivity of our results to changes in the parameters. The sensitivity factor was calculated as:

$$\frac{\Delta R}{\Delta P}$$

In this equation, $\Delta$R is the percent change in the result value and $\Delta$P is the percent change in the parameter value (S6 Data).

The parameters included in this analysis are: lifetime of clothing, production losses, washing frequency, percentage of clothing in-use, microfiber shed rate (machine and hand washing), WWTP microfiber retention rate, and washing machine ownership. Four parameters proved to be highly sensitive with a sensitivity factor of ≥1 or ≤-1 (S1 Table) while one parameter, washing machine ownership, was moderately sensitive. An alternative scenario analysis was performed below for each of these highly and moderately sensitive parameters (See S2 Table and S3 for results). Please see S1 Table for a summary of the results of the sensitivity analysis. See the supplementary excel file for the details of the sensitivity analysis.

S1 Table. Summary table of the sensitivity of each parameter on the results of landfill, incineration, waterbody, terrestrial environments, and total emissions.

|  | Sensitivity factor | | | | |
| --- | --- | --- | --- | --- | --- |
|  | Landfill | Incineration | Waterbodies | Terrestrial Environments | Total |
| Lifetime of clothing | 0.09 | 0.09 | 0.09 | 0.09 | 0.09 |
| Production losses | -0.25 | -0.25 | -0.25 | -0.25 | -0.25 |
| Washing frequency | 1.00 | 1.00 | 1.00 | 1.00 | 1.00 |
| Percentage of clothing in-use | 1.00 | 1.00 | 1.00 | 1.00 | 1.00 |
| Microfiber shed rate (machine and hand washing) | 1.00 | 1.00 | 1.00 | 1.00 | 1.00 |
| WWTP microfiber retention rate | 1.00 | 1.00 | -1.05 | 0.85 | 0.00 |
| Washing machine ownership | 0.45 | 0.47 | 0.41 | 0.45 | 0.43 |

**Alternative scenario analysis**

**Washing frequency**

Our washing frequency model produced an upper and lower bound of 6.1 and 10.1 washes per year. Under a 6.1 washes per year scenario, 2016 emissions decrease to 26.0 kt to landfills, 12.6 kt to incineration, 125.9 kt to waterbodies, and 106.9 kt to terrestrial environments. Total emissions for years 1950 to 2016 decrease to 0.4 Mt to landfills, 0.2 Mt to incineration, 2.2 Mt to waterbodies, and 1.4 Mt to terrestrial environments.

Under an 10.1 washes per year scenario, 2016 emissions increase to 43.1 kt to landfills, 20.9 kt to incineration, 208.5 kt to waterbodies, and 177.0 kt to terrestrial environments. Total emissions for years 1950-2016 increase to 0.7 Mt to landfills, 0.4 Mt to incineration, 3.6 Mt to waterbodies, and 2.4 Mt to terrestrial environments.

**Percentage of clothing in-use**

Available literature suggests that up to 30% of clothing may be considered inactive (1) whereas our model considers 25% of clothing to be inactive. For this reason, we used 30% inactive as our lower bound and moved the upper bound equidistant from the original value, resulting in an upper bound of 20%.

Under a 30% of clothing being inactive scenario, emissions for 2016 decrease to 32.3 kt to landfills, 15.7 kt to incineration, 156.1 kt to waterbodies, and 132.5 kt to terrestrial environments (S2 Table). Total cumulative emissions for years 1950-2016 decreased to 0.5 Mt to landfills, 0.3 Mt to incineration, 2.7 Mt to waterbodies, and 1.8 Mt to terrestrial environments (S3 Table).

Under a 20% of clothing being inactive scenario, emissions in 2016 increased to 36.9 kt to landfills, 17.9 kt to incineration, 178.4 kt to waterbodies, and 151.4 kt to terrestrial environments (S2 Table). The total cumulative emissions for years 1950-2016 increased to 0.6 Mt to landfill, 0.3 Mt to incineration, 3.1 Mt to waterbodies, and 2.1 Mt to terrestrial environments (S3 Table).

**Microfiber shed rate – machine washing**

The value for our microfiber shed rate was derived by taking the mean value across all studies that report microfiber shedding in units of mass shed per mass washed. For our alternative scenarios, we set the upper and lower bounds to be +30% and -30% of our current microfiber shed rate.

Under the lower bound scenario, emissions for 2016 decrease to 25.0 kt to landfills, 11.9 kt to incineration, 125.9 kt to waterbodies, and 103.2 kt to terrestrial environments (S2 Table). Total cumulative emissions for years 1950-2016 decrease to 0.4 Mt to landfill, 0.2 Mt to incineration, 2.2 Mt to waterbodies, and 1.4 Mt to terrestrial environments (S3 Table).

Under the upper bound scenario, emissions for 2016 increase to 44.2 kt to landfills, 21.6 kt to incineration, 208.5 kt to waterbodies, and 180.6 kt to terrestrial environments (S2 Table). Total cumulative emissions for years 1950-2016 increase to 0.7 Mt to landfills, 0.4 Mt to incineration, 3.5 Mt to waterbodies, and 2.4 Mt to terrestrial environments (S3 Table).

**Microfiber shed rate – hand washing**

To the best of our knowledge, there are currently no studies that measure microfiber shedding from hand washing garments. For this reason, the value used in our model is arbitrarily set at 50% of the machine washing shed rate and remains the largest source of uncertainty. For the alternative scenarios, we set the upper and lower bounds to be 25% and 75% of the machine washing shed rate.

Under the 25% shed rate scenario, emissions for 2016 decrease to 33.3 kt to landfills, 16.5 kt to incineration, 152.4 kt to waterbodies, and 135.5 kt to terrestrial environments (S2 Table). The total cumulative emissions for 1950-2016 decrease to 0.5 Mt to landfills, 0.3 Mt to incineration, 2.5 Mt to waterbodies, and 1.8 Mt to terrestrial environments (S3 Table).

Under the 75% shed rate scenario, emissions for 2016 increase to 35.8 kt to landfills, 17.1 kt to incineration, 182.0 kt to waterbodies, and 148.4 kt to terrestrial environments (S2 Table). Total cumulative emissions for years 1950-2016 increase to 0.6 Mt to landfills, 0.3 Mt to incineration, 3.3 Mt to waterbodies, and 2.1 Mt to terrestrial environments (S3 Table).

**WWTP microfiber retention rate**

The WWTP microfiber retention rates used for our model were collected from nine different studies. For the lower and upper bounds, we used the minimum and maximum value for primary and secondary treatment taken across all nine studies. For primary treatment, the minimum and maximum values are 32.4% and 99.0%, respectively. The minimum and maximum values for secondary treatment are 61.0% and 99.9%, respectively.

Under the lower bound scenario, emissions for 2016 are 19.7 kt to landfills, 10.7 kt to incineration, 240.1 kt to waterbodies, and 89.9 kt to terrestrial environments (S2 Table). Total cumulative emissions for years 1950-2016 are 0.3 Mt to landfills, 0.2 Mt to incineration, 3.8 Mt to waterbodies, and 1.3 Mt to terrestrial environments (S3 Table).

Under the upper bound scenario, emissions for 2016 are 37.9 kt to landfills, 17.9 kt to incineration, 150.9 kt to waterbodies, and 153.7 kt to terrestrial environments (S2 Table). Total cumulative emissions for years 1950-2016 are 0.6 Mt to landfills, 0.3 Mt to incineration, 2.7 Mt to waterbodies, and 2.1 Mt to terrestrial environments (S3 Table).

**Washing machine ownership**

Washing machine ownership is a moderately sensitive parameter as seen above in S1 Table. Due to the nature of the method from which the values were derived, there are no obvious upper and lower bounds to assign for the alternative scenarios. For this reason, we arbitrarily increased and decreased the existing values for each country’s percentage of the population owning washing machines by ± 20%. We set an upper and lower limit for the values at 100% and 0%.

In the first scenario, decreasing the annual percentage of the population owning washing machines by 20% resulted in a decrease of 2016 emissions to 30.9 kt to landfills, 15.0 kt to incineration, 148.1 kt to waterbodies, and 126.7 kt to terrestrial environments (S2 Table). Total cumulative emissions for years 1950-2016 decreased to 0.5 Mt to landfills, 0.3 Mt to incineration, 2.6 Mt to waterbodies, and 1.7 Mt to terrestrial environments (S3 Table).

In the second scenario, increasing the annual percentage of the population owning washing machines by 20% resulted in an increase of 2016 emissions to 36.0 kt to landfills, 17.3 kt to incineration, 176.9 kt to waterbodies, and 147.0 kt to terrestrial environments (S2 Table). Total cumulative emissions for years 1950-2016 increased to 0.6 Mt to landfills, 0.3 Mt to incineration, 3.1 Mt to waterbodies, and 2.0 Mt to terrestrial environments (S3 Table).

| Total (kt) | Lower Bound | 271.5 | 336.5 | 266.0 | 337.6 | 360.5 | 320.7 |
| --- | --- | --- | --- | --- | --- | --- | --- |
|  | Upper Bound | 449.5 | 384.5 | 454.9 | 383.3 | 360.5 | 377.2 |
| Terrestrial Environments (kt) | Lower Bound | 106.9 | 132.5 | 103.2 | 135.5 | 89.9 | 126.7 |
|  | Upper Bound | 177.0 | 151.4 | 180.6 | 148.4 | 153.7 | 147.0 |
| Waterbodies (kt) | Lower Bound | 125.9 | 156.1 | 125.9 | 152.4 | 240.1 | 148.1 |
|  | Upper Bound | 208.5 | 178.4 | 208.5 | 182.0 | 150.9 | 176.9 |
| Incineration (kt) | Lower Bound | 12.6 | 15.7 | 11.9 | 16.5 | 10.7 | 15.0 |
|  | Upper Bound | 20.9 | 17.9 | 21.6 | 17.1 | 17.9 | 17.3 |
| Landfill (kt) | Lower Bound | 26.0 | 32.3 | 25.0 | 33.3 | 19.7 | 30.9 |
|  | Upper Bound | 43.1 | 36.9 | 44.2 | 35.8 | 37.9 | 36.0 |
|  |  | Washing frequency | Percentage of clothing in-use | Microfiber shed rate (machine washing) | Microfiber shed rate (hand washing) | Microfiber retention rate (WWTP) | Washing machine ownership |

S2 Table. Results of the alternative scenario analysis on 2016 landfill, incineration, waterbody, terrestrial environments, and total emissions. The left column shows the parameters included in the analysis.

| Total (Mt) | Lower Bound | 4.3 | 5.3 | 4.3 | 5.1 | 5.6 | 5.0 |
| --- | --- | --- | --- | --- | --- | --- | --- |
|  | Upper Bound | 7.0 | 6.0 | 7.0 | 6.2 | 5.6 | 6.1 |
| Terrestrial Environments (Mt) | Lower Bound | 1.4 | 1.8 | 1.4 | 1.8 | 1.3 | 1.7 |
|  | Upper Bound | 2.4 | 2.1 | 2.4 | 2.1 | 2.1 | 2.0 |
| Waterbodies (Mt) | Lower Bound | 2.2 | 2.7 | 2.2 | 2.5 | 3.8 | 2.6 |
|  | Upper Bound | 3.6 | 3.1 | 3.5 | 3.3 | 2.7 | 3.1 |
| Incineration (Mt) | Lower Bound | 0.2 | 0.3 | 0.2 | 0.3 | 0.2 | 0.3 |
|  | Upper Bound | 0.4 | 0.3 | 0.4 | 0.3 | 0.3 | 0.3 |
| Landfill (Mt) | Lower Bound | 0.4 | 0.5 | 0.4 | 0.5 | 0.3 | 0.5 |
|  | Upper Bound | 0.7 | 0.6 | 0.7 | 0.6 | 0.6 | 0.6 |
|  |  | Washing frequency | Percentage of clothing in-use | Microfiber shed rate (machine washing) | Microfiber shed rate (hand washing) | Microfiber retention rate (WWTP) | Washing machine ownership |

S3 Table. Results of the alternative scenario analysis for total cumulative emissions to landfills, incineration, waterbodies, and terrestrial environments for years 1950-2016. The left column contains the parameters included in the analysis.

Fiber

production

Apparel

production

Microfiber

generation

Apparel

use

Machine

washing

Hand

washing

WWTP

No

WWTP

Other fiber

uses

Production

waste

End

-

of

-

life

apparel

Waterbodies

Terrestrial

Environments

Landfill

Incineration

Microfibers

in

sludge

Microfibers

in

wastewater

20%

~5

yrs

% varies

S1 Figure. Conceptual flow model of the generation, transport, and initial fate of synthetic microfibers from apparel washing.

S4 Table. Estimated percentages of microparticles removed during pretreatment, primary, secondary, and tertiary wastewater treatment. Each percentage is the mean of the values taken from the sources in the rightmost column.

| Treatment Level | % of microparticles removed | Sources |
| --- | --- | --- |
| Pretreatment | 44% | Murphy et al. (2015) |
|  |  | Michielssen et al. (2016) |
| Primary | 84% | Talvitie et al. (2016) |
|  |  | Murphy et al. (2015) |
|  |  | Lares et al. (2017) |
|  |  | Magnusson et al. (2016) |
|  |  | Michielssen et al. (2016) |
|  |  | Gies et al. (2018) |
| Secondary | 94% | Talvitie et al. (2016) |
|  |  | Murphy et al. (2015) |
|  |  | Carr et al. (2016) |
|  |  | Simon et al. (2018) |
|  |  | Lares et al. (2017) |
|  |  | Leslie et al. (2017) |
|  |  | Michielssen et al. (2016) |
|  |  | Gies et al. (2018) |
| Tertiary | 99% | Magnusson et al. (2016) |
|  |  | Michielssen et al. (2016) |

S5 Table. Per capita in-use stock of each region. The global average per capita stock is given in the rightmost column.

|  | North America (kt) | ROW (kt) | West Europe (kt) | East Europe (kt) | South Asia (kt) | China (with Hong Kong) (kt) | Global Average (kt) |
| --- | --- | --- | --- | --- | --- | --- | --- |
| 2016 | 62 | 26 | 41 | 35 | 17 | 51 | 26 |
| 2015 | 59 | 25 | 40 | 34 | 16 | 49 | 25 |
| 2014 | 56 | 25 | 38 | 34 | 15 | 46 | 24 |
| 2013 | 54 | 24 | 37 | 33 | 14 | 43 | 23 |
| 2012 | 53 | 23 | 36 | 31 | 13 | 40 | 22 |
| 2011 | 52 | 21 | 36 | 30 | 12 | 37 | 21 |
| 2010 | 53 | 19 | 37 | 28 | 11 | 35 | 20 |
| 2009 | 54 | 18 | 38 | 25 | 10 | 33 | 19 |
| 2008 | 56 | 16 | 40 | 23 | 10 | 31 | 18 |
| 2007 | 58 | 15 | 42 | 20 | 9 | 30 | 18 |
| 2006 | 57 | 13 | 42 | 17 | 8 | 28 | 17 |
| 2005 | 56 | 13 | 42 | 16 | 8 | 25 | 16 |
| 2004 | 55 | 12 | 41 | 14 | 8 | 23 | 15 |
| 2003 | 54 | 12 | 40 | 13 | 8 | 21 | 15 |
| 2002 | 53 | 11 | 38 | 12 | 8 | 19 | 14 |
| 2001 | 51 | 11 | 36 | 11 | 8 | 17 | 13 |
| 2000 | 51 | 11 | 34 | 11 | 8 | 15 | 13 |
| 1999 | 49 | 10 | 32 | 11 | 8 | 14 | 12 |
| 1998 | 47 | 9 | 30 | 10 | 7 | 12 | 11 |
| 1997 | 45 | 9 | 28 | 10 | 7 | 11 | 10 |
| 1996 | 43 | 8 | 27 | 11 | 6 | 9 | 10 |
| 1995 | 42 | 8 | 27 | 11 | 6 | 8 | 9 |
| 1994 | 40 | 7 | 26 | 12 | 6 | 7 | 9 |
| 1993 | 38 | 7 | 25 | 13 | 5 | 6 | 9 |
| 1992 | 37 | 7 | 24 | 14 | 5 | 6 | 8 |
| 1991 | 36 | 7 | 24 | 14 | 5 | 6 | 8 |
| 1990 | 36 | 7 | 23 | 14 | 5 | 5 | 8 |

External data file:

S1 Data: Global synthetic fiber production, percentage of synthetic fibers used for apparel production, mass of synthetic fibers present in apparel; Mass of fibers present in apparel by fiber type; End of life model; Mass of apparel reaching its end of life and annual in-use apparel stock spanning 1950-2016.

S2 Data: Annual Regional Apparel Stock

S3 Data: Percentages of households in each region owning washing machines from 1950-2016.

S4 Data: Percentages of each region's residential population connected to wastewater treatment from 1950-2016; Treatment type and sludge retention rates.

S5 Data: The percentages of sludge generated in each region that enters each initial compartment of emission (ie. waterbodies, terrestrial environments, landfill, and incineration).

S6 Data: Sensitivity Analysis
